# Rapid Aqueous Late‐Stage Radiolabelling of [GaF_3_(BnMe_2_‐tacn)] by ^18^F/^19^F Isotopic Exchange: Towards New PET Imaging Probes

**DOI:** 10.1002/anie.201802446

**Published:** 2018-04-26

**Authors:** Francesco M. Monzittu, Imtiaz Khan, William Levason, Sajinder K. Luthra, Graeme McRobbie, Gillian Reid

**Affiliations:** ^1^ School of Chemistry University of Southampton Southampton (UK) SO17 1BJ UK; ^2^ GE Healthcare The Grove Centre White Lion Road Amersham (UK) HP7 9LL UK

**Keywords:** fluorine-18, gallium, isotopic exchange, positron emission tomography, radiofluorination

## Abstract

A simple and rapid method for ^18^F radiolabelling of [GaF_3_(BnMe_2_‐tacn)] by ^18^F/^19^F isotopic exchange is described. The use of MeCN/H_2_O or EtOH/H_2_O (75:25) and aqueous [^18^F]F^−^ (up to 200 MBq) with heating (80 °C, 10 min) gave 66±4 % ^18^F incorporation at a concentration of 268 nm, and 37±5 % ^18^F incorporation at even lower concentration (27 nm), without the need for a Lewis acid promoter. A solid‐phase extraction method was established to give [Ga^18^F^19^F_2_(BnMe_2_‐tacn)] in 99 % radiochemical purity in an EtOH/H_2_O mixture.

Fluorine‐18 is the most widely utilised radioisotope in positron emission tomography (PET) imaging owing to its physical and nuclear characteristics: the short but manageable half‐life (ca. 110 min), the short positron linear range in tissue (2.3 mm), the lack of side emission (97 % decay by positron emission), the low energy of the positron (*E* ${}_{\beta {}_{\max}}$ =635 keV), and the wide availability of cyclotrons for its production. The ^18^F half‐life is sufficient to allow for a certain degree of manipulation of the synthesis, provided that the radiolabelling occurs in the later stages of the synthesis (ideally in the final step). The most commonly used PET radiotracers are organic molecules where the radioactive fluorine atom is attached to a carbon atom. Their generation often requires multistep syntheses and/or purification after the labelling step, which can be time‐consuming and inefficient. This has driven recent work by several groups to investigate the ^18^F radiolabelling properties of inorganic molecules, where the strong bond between (typically) a main‐group element and fluorine can be exploited to enable fast late‐stage radiolabelling. Other important aspects to be considered are pH tolerance and the temperature required for radiolabelling as these will significantly influence the compatibility with biomolecules. An ideal target for ^18^F radiolabelling would be a method consisting of a single step where the [^18^F]F^−^ target water is introduced directly without further purification, at very low precursor concentration (e.g., 10 nm), without the need for post‐labelling purification, giving a product with high molar activity that is stable in the formulation matrix.

A recent review from Gabbaï and co‐workers describes some of the key advances in the development of Group 13 element based tracers towards PET applications.^1^ Within the Group 13 elements, boron has the highest bond dissociation energy with fluorine (>730 kJ mol^−1^),^2^ and after carbon, it has been the most studied element for PET applications. Several different types of molecules have been successfully radiolabelled with ^18^F, including aryl trifluoroborates,^3^, ^4^, ^5^ zwitterionic onium trifluoroborates,^6^, ^7^, ^8^ and BODIPY‐based dyes.^9^,^10^ Typically, radiofluorination is achieved by either converting a boronic ester moiety into a fluoroborate species or by an isotopic exchange reaction. Very recently, the development of [^18^F]BF_4_^−^ and [^18^F]CF_3_SO_3_^−^ as PET probes for imaging the sodium iodide symporter has been reported.^11^, ^12^, ^13^

Work on the “Al‐F” system developed by McBride and co‐workers demonstrated that radiofluorination can be achieved by heating AlCl_3_, [^18^F]F^−^, and a pentadentate NOTA‐derived ligand together in aqueous solution at pH 3.9–4.2 and 100 °C (NOTA=1,4,7‐triazacyclononane‐1,4,7‐triacetic acid).^14^ This was an important breakthrough, providing the first example of a metal chelate system for [^18^F]F^−^ capture in water, and exploiting coordination chemistry to determine fluoride incorporation. A related gallium species was reported subsequently; in this case, the [Ga^18^F(L)] moiety (L=1‐benzyl‐1,4,7‐triazacyclononane‐4,7‐dicarboxylate) was formed readily by radiofluorination of the preformed chloride complex [GaCl(L)] in aqueous MeCN under mild conditions. This ^18^F‐radiolabelled complex is stable to at least pH 6, but unlike the Al‐F system, exhibits reduced stability in phosphate buffered saline (PBS) and human serum albumin (HSA) at pH 7.4.^15^ This instability at higher pH was attributed, at least in part, to the lower stability of the carboxylate bonds to Ga^III^ versus Al^III^.

In 2014, we reported a series of trifluoride complexes of aluminium, gallium, and indium with neutral tridentate tacn‐based macrocycles, including [MF_3_(BnMe_2_‐tacn)] (M=Al, Ga, In; BnMe_2_‐tacn=1‐benzyl‐4,7‐dimethyl‐1,4,7‐triazacyclononane), as potential [^18^F]F^−^ carrier molecules. Notably, these trifluoro complexes are extremely stable in water.^16^ Furthermore, we demonstrated that the trichloro analogue, [GaCl_3_(BnMe_2_‐tacn)], can be radiofluorinated easily at room temperature and close to neutral pH, by treating an MeCN/H_2_O solution of the complex with 2.99 equiv of aqueous KF doped with [^18^F]KF (100–500 MBq). The resulting [Ga^18^F^19^F_2_(BnMe_2_‐tacn)] complex shows very high radiochemical stability in PBS at pH 7.4 (with a radiochemical purity of 98 % after 120 min).

In the fluorination of the metal trichloro species, the strength of the M−F bonds being formed undoubtedly provides a significant thermodynamic driving force for the rapid introduction of F^−^. It is also clear that the stability of the resultant radiofluorinated metal complexes in competitive media (PBS or serum) is also subtly dependent upon the choice of metal ion acceptor and any co‐ligands present in the metal coordination sphere.^15^

Whilst radiofluorination was readily achieved for [MCl_3_(BnMe_2_‐tacn)] (M=Ga, Al) at 2.6 μm concentration,^16^,^17^ to offer real prospects for the development of a viable imaging probe for PET based upon this system, it is desirable for the ^18^F incorporation to be efficient at lower, that is, nanomolar concentration. However, we found that [MCl_3_(BnMe_2_‐tacn)] did not undergo radiofluorination at 260 nm (0.1 mg mL^−1^) concentration. We surmised that this could be due to the hydrolytic sensitivity of the M−Cl groups in [MCl_3_(BnMe_2_‐tacn)], resulting in competition between slow hydrolysis and Cl/F exchange under the labelling conditions.^16^

In view of the apparent resistance of [GaF_3_(BnMe_2_‐tacn)] to hydrolysis, we first sought to probe the kinetic stability of this (inactive) trifluoro complex in aqueous solution under a variety of conditions, before exploring the possibility of using ^18^F/^19^F isotopic exchange reactions to produce the radiofluorinated product more conveniently and, preferably, using less material.

A more convenient synthesis of (inactive) [Ga^19^F_3_(BnMe_2_‐tacn)] was established as an alternative to the hydrothermal method that we described previously.^16^ The new method is based on the direct reaction of the molecular [GaF_3_(dmso)(OH_2_)_2_] complex with BnMe_2_‐tacn in CH_2_Cl_2_ at room temperature, giving the characteristic IR and ^1^H, ^19^F, and ^71^Ga NMR spectroscopic signatures (see the Supporting Information, Figures S1–S3); [Ga^19^F_3_(BnMe_2_‐tacn)] was then isolated in 87 % yield. Electrospray ionization mass spectrometry in the positive‐ion mode (MeCN/H_2_O) gave *m*/*z* values consistent with [GaF_3_(BnMe_2_‐tacn)+Li]^+^ and [GaF_2_(BnMe_2_‐tacn)]^+^ (Figure S4); the strong affinity of [MF_3_(R_3_‐tacn)] to alkali‐metal cations has been demonstrated previously.^18^

Solution ^19^F NMR studies confirmed that [GaF_3_(Me_3_‐tacn)] is very stable in water (spectrum unchanged) at elevated temperature (80 °C), even after several hours. This is in contrast to the stability of [GaCl_3_(RMe_2_‐tacn)] (R=Me or Bn), which hydrolyses within minutes when small amounts of water are added to a solution of the complex in MeCN at room temperature.^16^ The trifluoro complex also shows very good pH tolerance, with no detectable degradation between pH 4 and 11, and similarly, the spectra were unchanged upon addition of a tenfold excess of (potentially) competitive chloride, carbonate, acetate, or phosphate anions (as sodium salts in H_2_O) to [GaF_3_(RMe_2_‐tacn)].

This stability of [Ga^19^F_3_(RMe_2_‐tacn)] (R=Bn, Me) in water led us to consider the prospect of radiolabelling [Ga^19^F_3_(BnMe_2_‐tacn)] by ^18^F/^19^F isotopic exchange. The trifluoro complex should be a more convenient precursor owing to its ease of handling, together with its much longer shelf‐life compared to that of [GaCl_3_(BnMe_2_‐tacn)]. Most importantly, we surmised that [Ga^19^F_3_(BnMe_2_‐tacn)] might facilitate radiofluorination at lower concentration as the likelihood of hydrolysis is considerably lower.

Previous work by the group of Schirrmacher^19^ on silicon fluoride based systems as well as by the groups of Blower,^20^ Gabbaï,^6^ and Perrin^21^ on boron fluoride systems had demonstrated the value of ^18^F/^19^F isotopic exchange in a number of PET tracers. A Lewis acid promoter such as SnCl_4_ is sometimes required to activate the fluorine–element bond to facilitate [^18^F]F^−^ incorporation in these systems.

Initial radiofluorination experiments were performed at a [Ga^19^F_3_(BnMe_2_‐tacn)] concentration of 2.6 μm in MeCN/H_2_O, using [^18^F]F^−^ target water directly (up to 200 MBq). The solvent conditions (MeCN/H_2_O, EtOH/H_2_O) and ratio, as well as the temperature (25 °C and 80 °C), were varied to establish the effect on the [^18^F]F^−^ incorporation (Table 1).

**Table 1:** Conditions used for ^18^F/^19^F radiofluorination experiments.^[a]^

[GaF_3_(BnMe_2_‐tacn)] [mg]	Scale [nmol]	Organic solvent	Organic solvent/ H_2_O ratio	*T* [°C]	*t* [min]	RCY^[b]^ [%]
1	2680	MeCN	8:92	25	45	4±2
1	2680	MeCN	8:92	80	30	18±4
1	2680	MeCN	50:50	80	60	23±4
1	2680	MeCN	75:25	80	10	73±4
0.1	268	MeCN	75:25	25	80	8±4
0.1	268	MeCN	75:25	80	10	66±4
0.01	27	MeCN	75:25	80	10	37±5
1	2680	EtOH	75:25	80	10	81±1
0.1	268	EtOH	75:25	80	10	50±4

[a] All experiments were performed at least three times. [b] Radiochemical yields (RCYs) determined by HPLC analysis.

The incorporation of [^18^F]F^−^ into [GaF_3_(BnMe_2_‐tacn)] occurred readily and reproducibly even at room temperature in 75:25 MeCN/H_2_O, with [Ga^18^F^19^F_2_(BnMe_2_‐tacn)] being the major radiolabelled species, the only other being unreacted [^18^F]F^−^. Moreover, heating the unbuffered mixture to 80 °C for 10 min also led to high [^18^F]F^−^ incorporation (typically between 65–73 % using both 2.68 μm and 268 nm solutions of the complex), without the need for a Lewis acid promoter. Radio‐HPLC traces are shown in Figure 1 and in the Supporting Information, Figures S5 and S6. The identity of the radiolabelled product was confirmed with the corresponding UV traces (Figures S11–S13). Using the relatively low‐activity [^18^F]F^−^ employed in this work (ca. 200 MBq), the molar activity^22^ determined for the 27 nm precursor concentration was ca. 675 MBq μmol^−1^.

**Figure 1:**
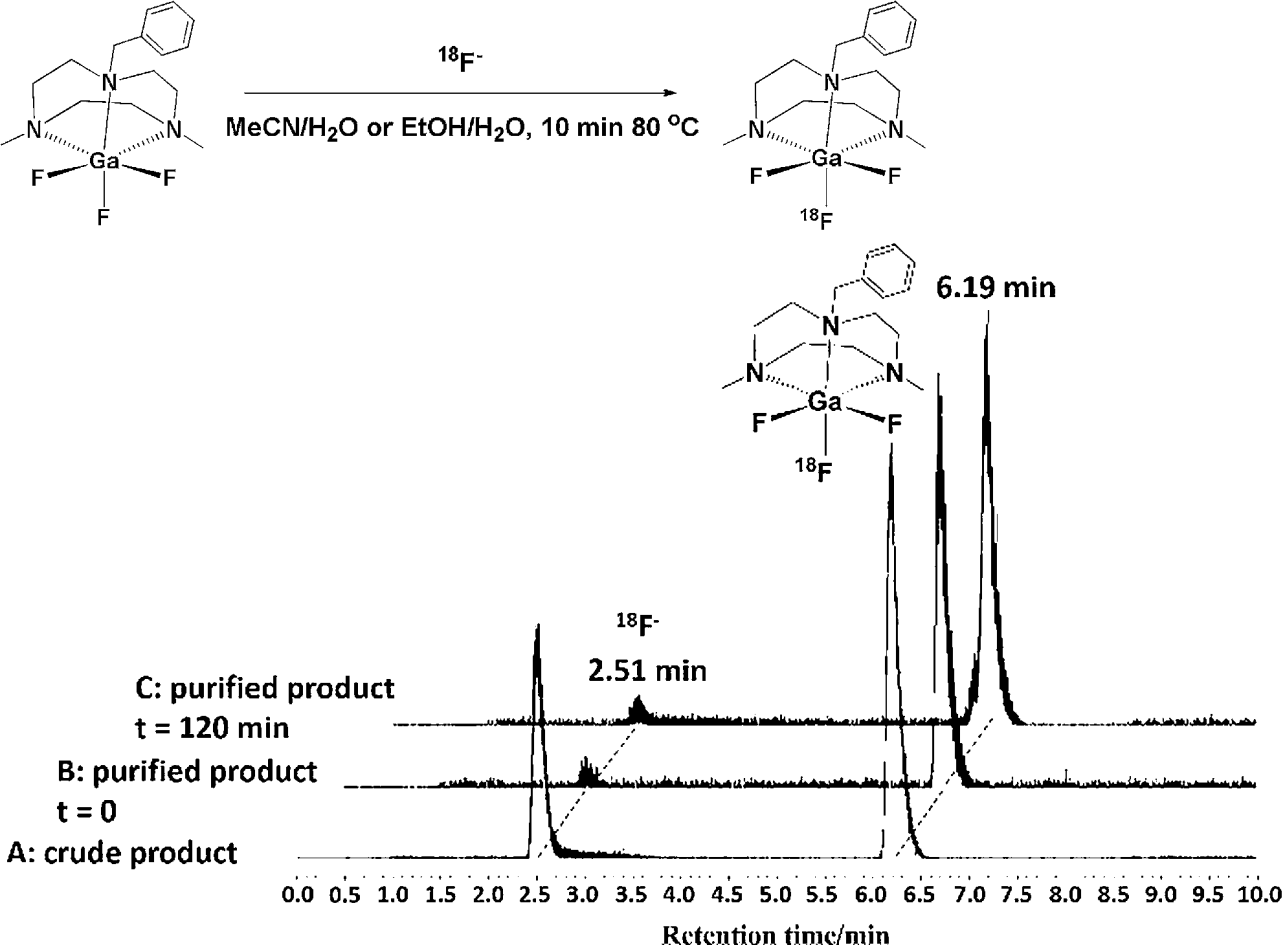
A) Radio‐HPLC chromatogram of the crude product. Peak 1: *R*_t_=2.51 min, 35 % (^18^F^−^). Peak 2: *R*_t_=6.19 min, 65 % ([Ga^18^F^19^F_2_(BnMe_2_tacn)]). B) Radio‐HPLC chromatogram of the purified product eluted from an HLB cartridge (formulated in 20 % EtOH/H_2_O). Peak 1: *R*_t_=2.49 min, 1 % (^18^F^−^). Peak 2: *R*_t_=6.18 min, 99 % ([Ga^18^F^19^F_2_(BnMe_2_tacn)]). C) Radio‐HPLC chromatogram of the purified product after 120 min (formulated in 20 % EtOH/H_2_O). Peak 1: *R*_t_=2.53 min, 12 % (^18^F^−^). Peak 2: *R*_t_=6.20 min, 88 % ([Ga^18^F^19^F_2_(BnMe_2_tacn)]).

A simple purification procedure using a hydrophilic lipophilic balanced (HLB) solid‐phase extraction (SPE) cartridge has also been established (see the Experimental Section), which gave the desired complex with a radiochemical purity of about 99 %.

This method was also adopted successfully at an even lower (27 nm) precursor complex concentration, resulting in 37±5 % ^18^F incorporation under similar labelling conditions (Table 1). This value corresponds to a decrease in the concentration of two orders of magnitude compared to the radiofluorination of [MCl_3_(BnMe_2_‐tacn)] by Cl/F exchange reported previously.

The radiochemical stability of purified [Ga^18^F^19^F_2_(BnMe_2_‐tacn)] was also monitored over time (typically 2–3 h) for a series of samples. After SPE purification with an HLB cartridge, we found that the RCP had decreased to between 88 % and 77 % after 120 min at room temperature through loss of [^18^F]F^−^ from the radiolabelled product. An alternative purification procedure based on the use of an HLB cartridge followed by an alumina cartridge (at *t*=0 min) gave similar results, whereas cooling the purified [Ga^18^F^19^F_2_(BnMe_2_‐tacn)] solution to −20 °C led to an RCP of approximately 93 % after 4 h (Figure S7).

As the Δ*G* value for the isotopic exchange is approximately zero, and some (ca. 4 %) [^18^F]F^−^ incorporation is seen at room temperature (with much higher incorporation at 80 °C; Table 1), the following equilibrium reaction must apply:

(1) $$\left[\mathrm{Ga}{}^{18}F{}^{19}F{}_{2}\left(\mathrm{BnMe}{}_{2}\text{-}\mathrm{tacn}\right)\right]+{}^{19}F{}^{-}\vec{\leftarrow }\left[\mathrm{Ga}{}^{19}F{}_{3}\left(\mathrm{BnMe}{}_{2}\text{-}\mathrm{tacn}\right)\right]+{}^{18}F{}^{-}$$

The effect of added fluoride or chloride was tested by the addition of either a 10 % K^19^F solution or a 0.9 % saline solution to purified [Ga^18^F^19^F_2_(BnMe_2_‐tacn)] at 25 °C. These had no significant effect on the RCP over about 2 h. These results are consistent with the isotopic exchange proceeding through a dissociative mechanism; that is, it is first order in [GaF_3_(BnMe_2_‐tacn)] and independent of the concentration of the entering ligand. Performing the radiofluorination in DMSO, a much more competitive (strongly coordinating) solvent, leads to a significant drop in the RCY, which is also consistent with a predominantly dissociative mechanism at the distorted octahedral Ga^III^ complex that proceeds via a five‐coordinate intermediate.^23^

Testing the stability of the radiolabelled product in 90 % human serum albumin (HSA)/10 % EtOH gave RCPs of 97 % at *t*=0 min and 83 % at *t*=120 min (Figure S8). The possibility of radiolysis leading to a decrease in RCP over time was also investigated, both by formulating the purified radiolabelled product in 10 % EtOH/PBS and by the addition of ascorbic acid to the purified radiolabelled product; neither had any appreciable effect (Figures S9 and S10).

Performing the radiofluorination of [Ga^18^F^19^F_2_(BnMe_2_‐tacn)] in 75:25 EtOH/H_2_O under the same conditions (80 °C/10 min) also led to high [^18^F]F^−^ incorporation (Table 1) with an RCP of 82 % after 2 h.

In summary, we have described the first ^18^F/^19^F isotopic exchange on a metal chelate system. The new method leads to high ^18^F incorporation by using [^18^F]F^−^ target water directly from the cyclotron and without the need for a Lewis acid promoter. Furthermore, we have shown that the method also allows for the concentration of the [GaF_3_(BnMe_2_‐tacn)] used for the radiofluorination to be scaled down by at least two orders of magnitude (27 nm, 0.01 mg), which represents a very significant decrease in the quantity of material needed compared to the Cl^−^/^18^F^−^ exchange reaction that we reported previously.^16^

The results reported here suggest that [Ga^19^F_3_(BnMe_2_‐tacn)] offers a promising basis for the development of new PET probes. Future work will explore this system further 1) by using computational and experimental work to determine the effects that parameters such as the choice of the Group 13 metal and altering the steric protection around the M−F groups have on the ^18^F incorporation, and 2) through the conjugation of peptides (via the benzyl pendant group) to the most promising candidates to evaluate them as radiotracers in biodistribution studies.

## Conflict of interest

The authors declare no conflict of interest.

## Supporting information

As a service to our authors and readers, this journal provides supporting information supplied by the authors. Such materials are peer reviewed and may be re‐organized for online delivery, but are not copy‐edited or typeset. Technical support issues arising from supporting information (other than missing files) should be addressed to the authors.

Supplementary
