# Supplementary material for: Rapid Aqueous Late‐Stage Radiolabelling of [GaF3(BnMe2‐tacn)] by 18F/19F Isotopic Exchange: Towards New PET Imaging Probes
Source: Angew Chem Int Ed Engl. 2018 Apr 26;57(22):6658–61. doi: 10.1002/anie.201802446 (PMC6055623; doi:10.1002/anie.201802446)
Supplement: Supplementary file 1 — Supplementary [file ANIE-57-6658-s001.pdf]

Angewandte Chemie
Eine Zeitschrift der Gesellschaft Deutscher Chemiker

Supporting Information

Rapid Aqueous Late-Stage Radiolabelling of [GaF_3($BnMe_2$-tacn)] by $^{18}F/^{19}F$ Isotopic Exchange: Towards New PET Imaging Probes

*Francesco M. Monzittu, Imtiaz Khan, William Levason, Sajinder K. Luthra, Graeme McRobbie, and Gillian Reid**

anie_201802446_sm_miscellaneous_information.pdf

Supporting Information

Experimental Section

[GaF_3(dmso)(OH_2)$_2$] was prepared according to literature methods.[1]

[GaF_3($BnMe_2$-tacn)]: [GaF_3(dmso)(OH_2)$_2$] (0.040 g, 0.16 mmol) was suspended in 5 mL of CH_2Cl_2. A solution of $BnMe_2$-tacn (0.041 g; 0.016 mmol) in 3 mL of CH_2Cl_2 was added. After 10 mins. the solution became clear. The reaction mixture was left stirring for a further hour at room temperature. The solvent was then reduced to ~ 4 mL and 15 mL of hexane was added, causing the precipitation of a white solid, which was filtered, washed with hexane (5 mL) and dried *in vacuo* (0.054 g; 87 %). Spectroscopic data match that reported.[14] ^{1}H NMR (D_2O, 298 K): 7.52-7.45 (m, [5H], ArH), 4.16 (s, [2H], Ar-CH_2), 3.30-3.22 (m, [2H], tacn-CH_2), 3.06-2.90 (m, [8H], tacn-CH_2), 2.72 (s, [6H], CH_3), 2.60-2.53 (m, [2H], tacn-CH_2). ^{19}F{^{1}H} NMR (D_2O, 298 K): −172.5 (br). ^{71}Ga NMR (D_2O, 298 K): +44.3 (br). ES^+ MS (MeCN/H_2O): *m/z* = 380 ([GaF_3($BnMe_2$-tacn)+Li]$^+$), 354 ([GaF_2($BnMe_2$-tacn)]$^+$).

HPLC purification: Column: Waters XBridge Prep Shield RP18, 5 um, 10 x 100 mm (p/n 186003258, s/n 115/123411KK01); Dionex Ultimate 3000 pump; Knauer Smartline 2500 UV detector. [GaF_3($BnMe_2$-tacn)] (7.3 mg) was dissolved in 4 mL of water and injected onto the prep. HPLC loop (mobile phase A = 100% water; B = 100% MeCN). Flow rate 3 mL min^{-1}. Gradient 0 to 10 min (0-10% B), 10-15 min (10-90% B), 15-20 min (90% B), 20-25 min (90-2% B). 6.0 mg of purified [GaF_3($BnMe_2$-tacn)] was obtained.

^{18}F/^{19}F Isotopic Exchange Radiolabelling Procedure: In a typical experiment, [GaF_3($BnMe_2$-tacn)] (1 mg, 2.68 μmol, 0.1 mg, 268 nmol or 0.01 mg, 27 nM) was dissolved in MeCN (n = 18) or EtOH (n = 5) (0.75 mL). To this solution was added 0.25 mL of an aqueous solution containing [^{18}F]F^- (10-200 MBq) and the vial was heated to 80 °C for 10 mins. The crude reaction solution was diluted with water (20 mL) so that approximately 10% of the solvent composition was organic. A small sample (~ 100 uL) of the diluted crude reaction solution was removed for analysis by analytical HPLC, which confirmed the percentage incorporation of ^{18}F into the metal complex (based upon integration of the radio peaks). [Note that 8% ^{18}F incorporation was observed when the radiolabelling experiment was performed at 2.36 μM concentration in MeCN/H_2O (75:25) at room temperature].

SPE Purification Protocol: The diluted reaction mixture was then trapped on a HLB cartridge, washed with water (5 mL x 3) to remove the $^{18}F^-$ and residual MeCN and eluted from the cartridge with ethanol (1 mL) into water to result in a formulated product in 80:20 water:EtOH. The formulated product was analysed by HPLC at t = 0 and various time intervals up to 240 mins. Similar procedures were used to test stability in pH 7.4 PBS and pH 7.5 HSA, giving 90:10 solutions that were monitored over 120 mins.

Experiments were analysed on an Agilent 1290 HPLC system with an Agilent 1260 DAD UV detector (G4212B). Dionex Chromeleon 6.8 Chromatography data recording software was used to integrate the radiochemical peak areas.

Analytical HPLC method: Column: Phenomenex Luna 5 um C18(2) 250 x 4.6 mm. Mobile phase A = 10 mM ammonium acetate, B = MeCN. Flow rate 1 mL min^{-1}. Gradient 0-15 min (10-90 % B), 15-20 min (90 % B), 20-21 min (90-10 % B), 21-26.5 min (10 % B).

In a typical experiment: Crude - Peak 1: Rt = 2.51 min ($^{18}F^-$). Peak 2: Rt = 6.19 min (complex).

Addition of saline, KF, OH^{-} solutions to the SPE purified radiolabelled product: Radiolabelling experiment and SPE purification protocol performed as reported above (0.1 mg of [GaF_3($BnMe_2$-tacn)], MeCN). The product was eluted from the cartridge with 1.6 mL of EtOH and this volume divided into three vials; a 0.9 % saline solution, a 10 % KF solution and a 20% NaOH solution were added (one for each vial). The RCP of the solutions was checked after 120 minutes by analytical HPLC giving RCP values of 80, 82 and 83%, respectively.

$^{18}F/^{19}F$ Isotopic Exchange Radiolabelling Procedure in dmso: [GaF_3($BnMe_2$-tacn)] (0.1 mg, 268 nmol) was dissolved in dmso (0.75 mL). To this solution was added 0.25 mL of an aqueous solution containing [^{18}F]F^{-} (62 MBq) and the vial was heated to 80 °C for 10 mins. The crude reaction was diluted with 1 mL of water. A small sample (~ 100 uL) of the diluted crude reaction solution was removed for analysis by analytical HPLC, giving a RCY of 14%.

[1] R. Bhalla, J. Burt, A. L. Hector, W. Levason, S. K. Luthra, G. McRobbie, F. M. Monzittu, G. Reid, *Polyhedron*, **2016**, *106*, 65.

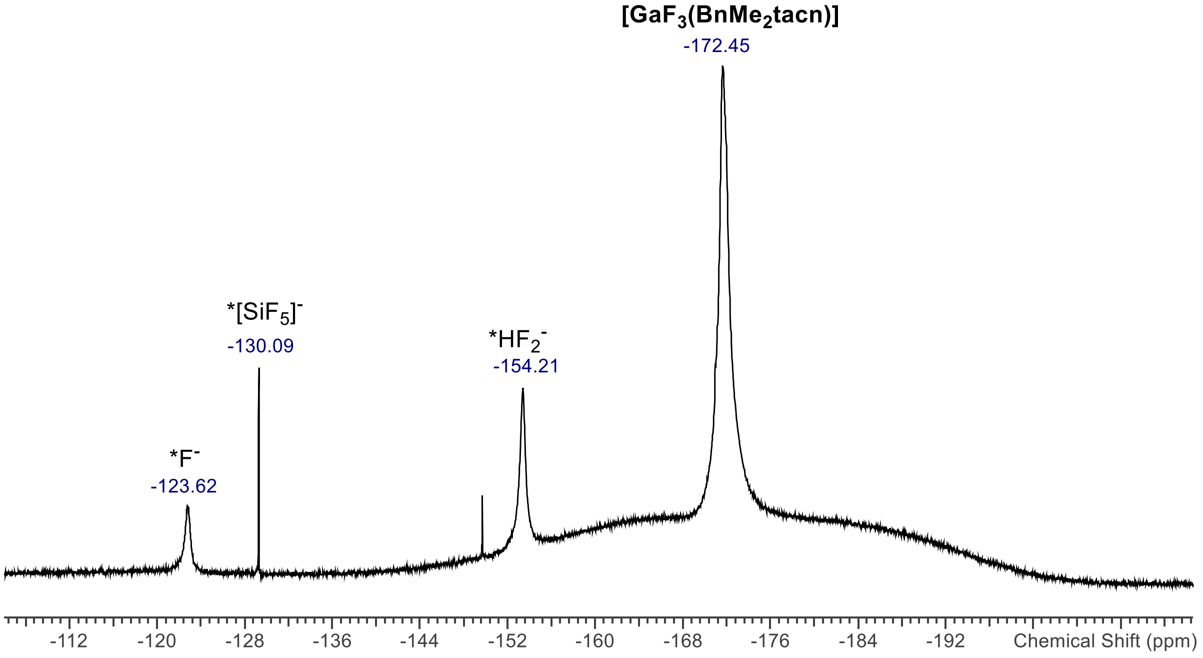

Figure S1. $^{19}F\{^{1}H\}$ NMR spectrum of [GaF_3($BnMe_2$-tacn)] in D_2O.

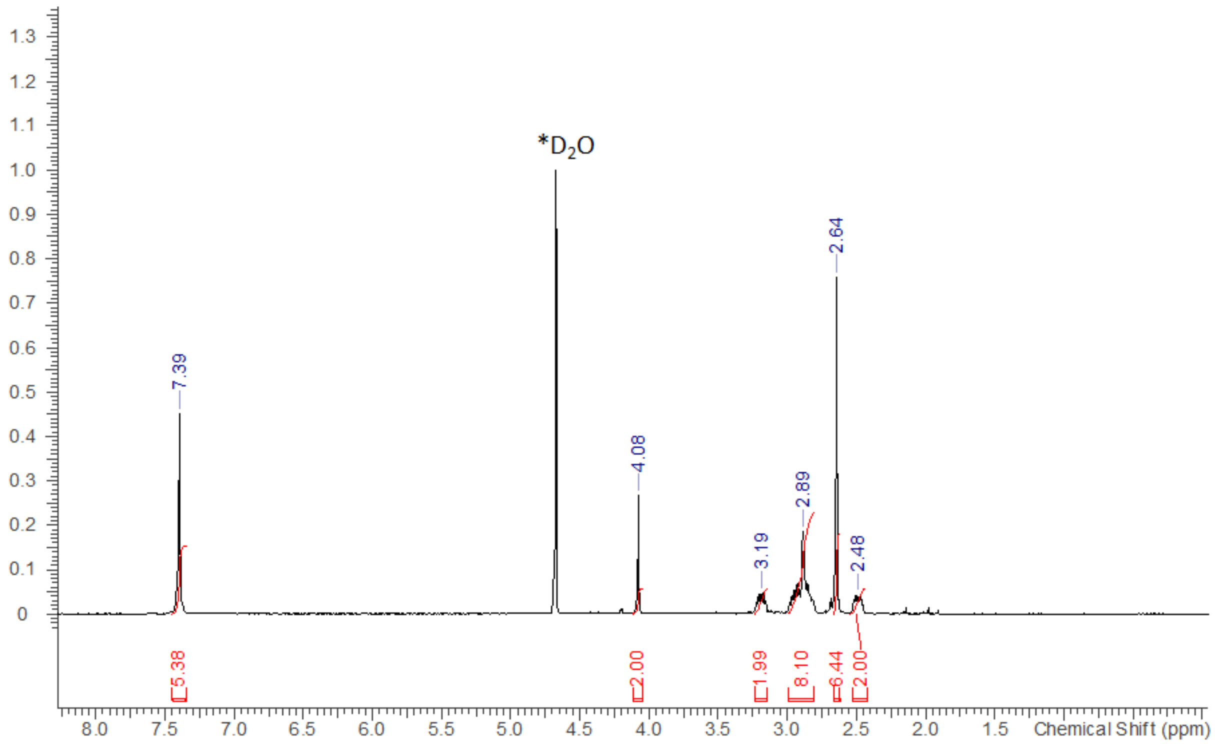

Figure S2. ^{1}H NMR spectrum of $[GaF_3(BnMe_2\text{-tacn})]$ in D_2O.

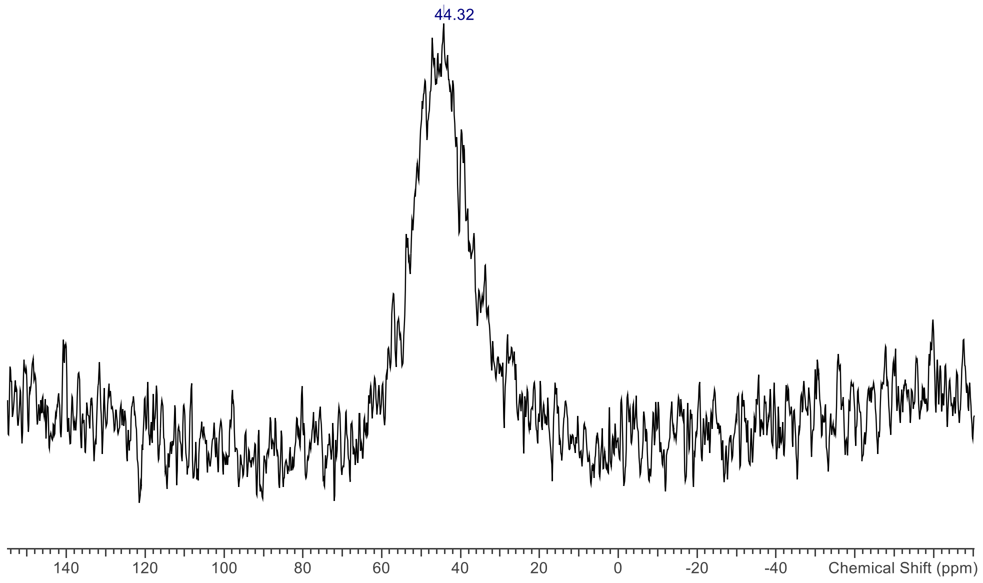

Figure S3. ^{71}Ga NMR spectrum of $[GaF_3(BnMe_2\text{-tacn})]$ in D_2O.

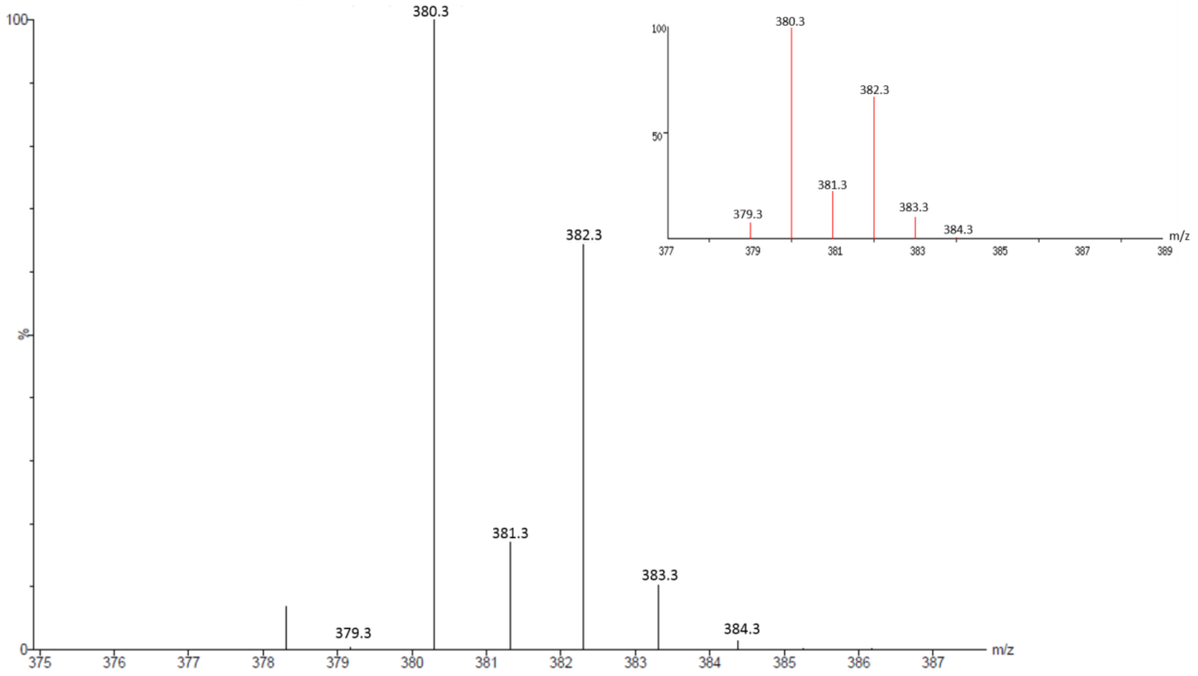

Figure S4. ES$^+$ mass spectrum of $[GaF_3(BnMe_2\text{-tacn})+Li]^+$ (left) and the predicted isotope pattern for $[GaF_3(BnMe_2\text{-tacn})+Li]^+$ (right).

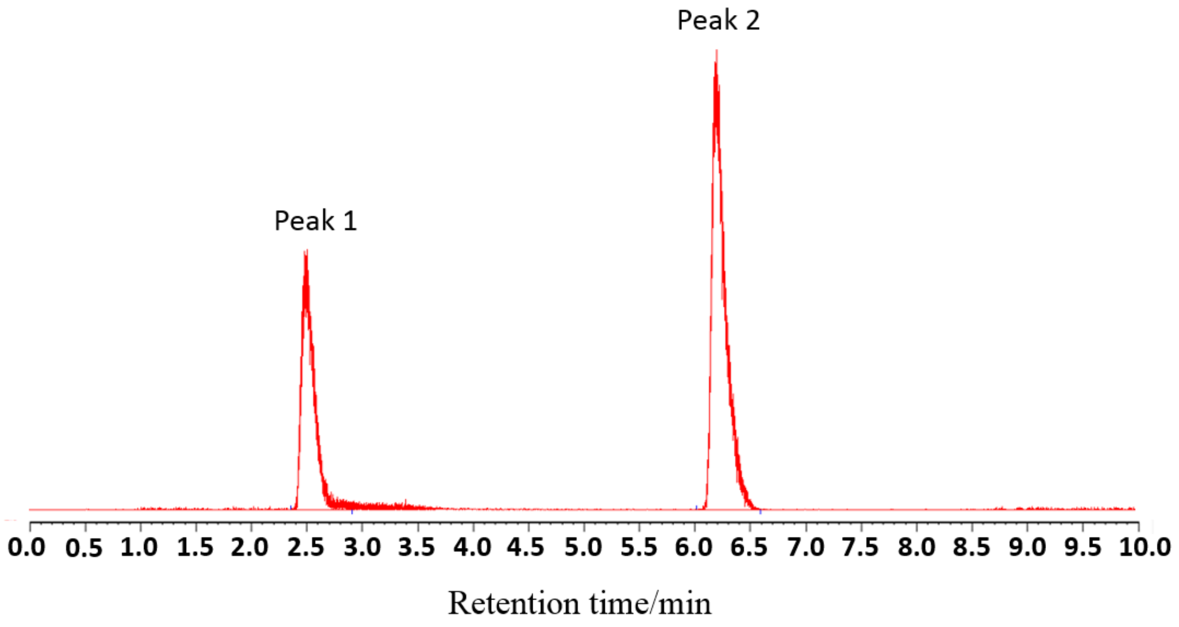

Figure S5. Radio-HPLC chromatogram of the crude product from radiofluorination of $[GaF_3(BnMe_2\text{-tacn})]$ (0.1 mg, 268 nmol) in 75%/25% CH_3CN/H_2O at 80 °C for 10 mins. Peak 1: Rt = 2.51 min 35% ($^{18}F^-$). Peak 2: Rt = 6.19 min 65% ($[Ga^{18}F^{19}F_2(BnMe_2\text{-tacn})]$).

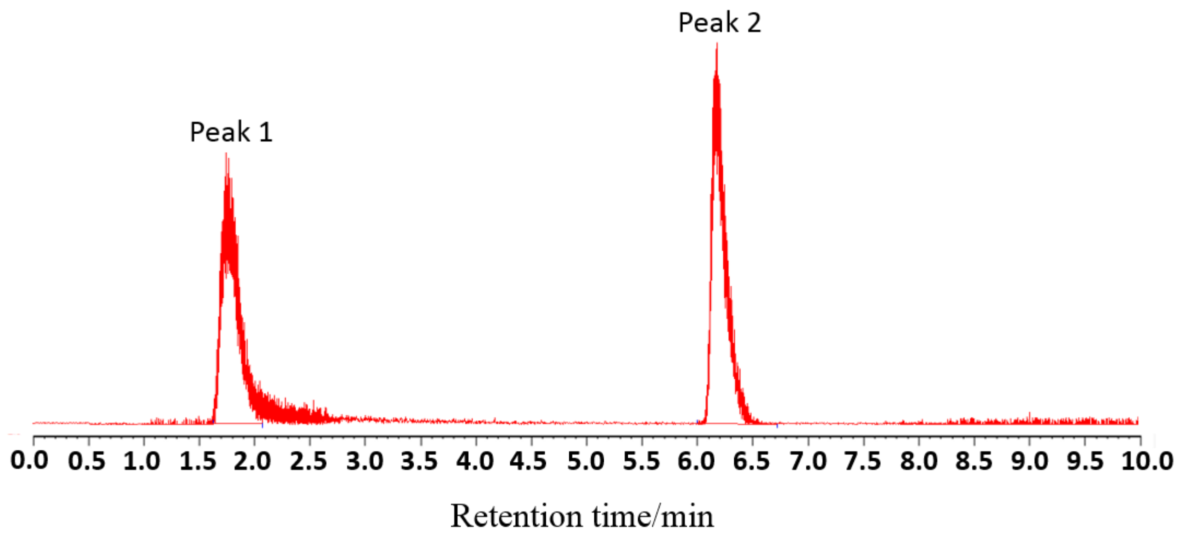

Figure S6. Radio-HPLC chromatogram of the crude product from radiofluorination of [GaF_3($BnMe_2$-tacn)] (0.1 mg, 268 nmol) in 75%/25% EtOH/H_2O at 80 °C for 10 mins. Peak 1: Rt = 2.57 min 46% ($^{18}F^-$). Peak 2: Rt = 6.16 min 54% ([$Ga^{18}F^{19}F_2$($BnMe_2$-tacn)]).

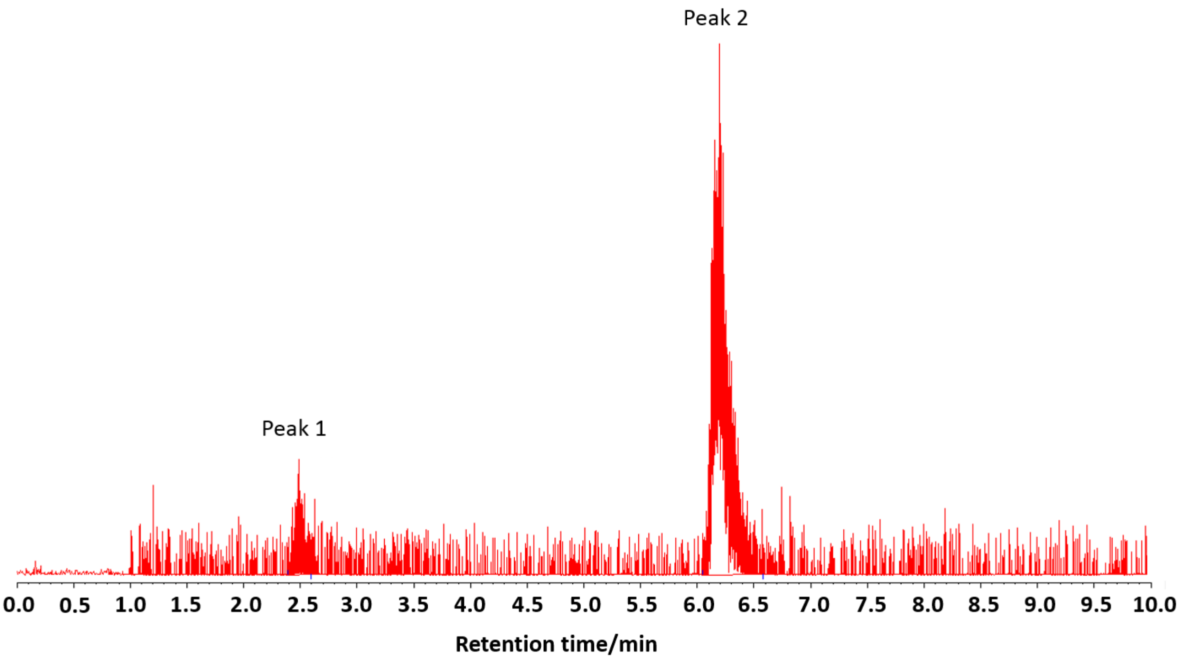

Figure S7. Radio-HPLC chromatogram of the purified [$Ga^{18}F^{19}F_2$($BnMe_2$tacn)] formulated in 20 % EtOH/water kept at −20 °C for 240 min. Peak 1: Rt = 2.51 min 6% ($^{18}F^-$). Peak 2: Rt = 6.21 min 94% ([$Ga^{18}F^{19}F_2$($BnMe_2$-tacn)]).

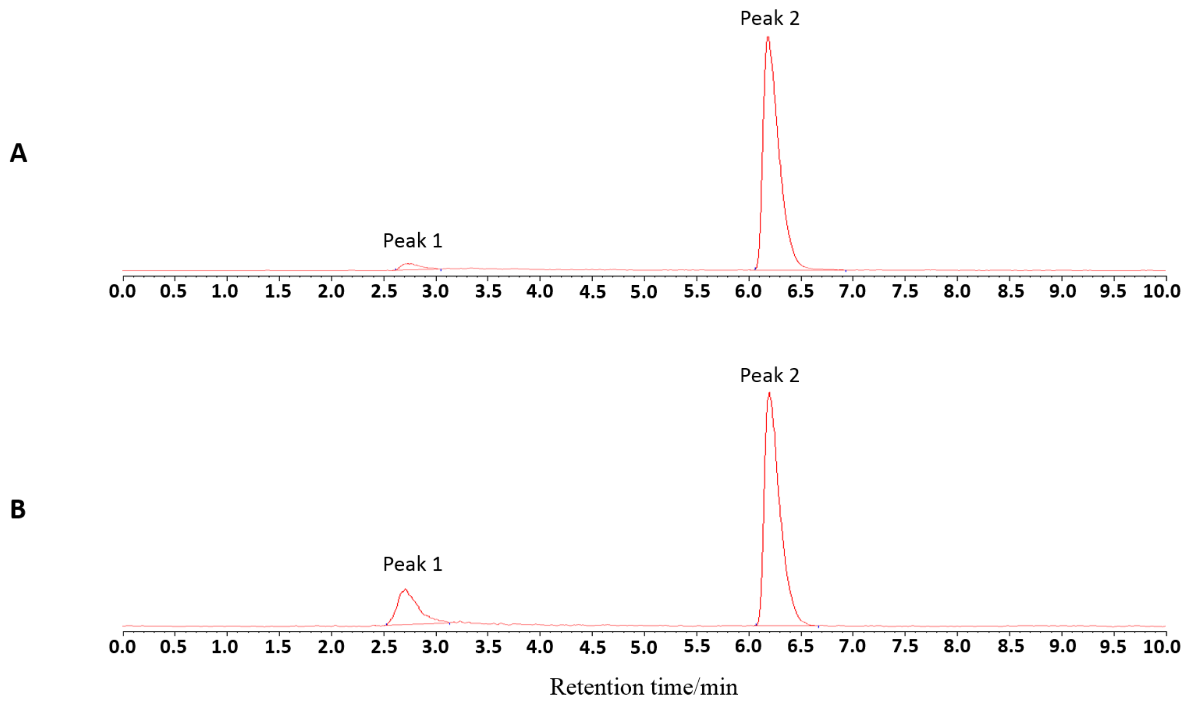

Figure S8. % RCP trend over time for the radio-product from [$GaF_3(BnMe_2$-tacn)] (0.1 mg, 268 nmol) and [^{18}F]F^- in 75%/25% $MeCN/H_2O$ at 80 °C for 10 mins., purified through a HLB cartridge and formulated in 10% EtOH/HSA. **A**: radio-HPLC chromatogram at t = 0; Peak 1: Rt = 2.74 min 3% ($^{18}F^-$). Peak 2: Rt = 6.18 min 97% ([$Ga^{18}F^{19}F_2(BnMe_2tacn)$]). **B**: radio-HPLC chromatogram at t = 120; Peak 1: Rt = 2.71 min 17% ($^{18}F^-$). Peak 2: Rt = 6.19 min 83% ([$Ga^{18}F^{19}F_2(BnMe_2$-tacn)]).

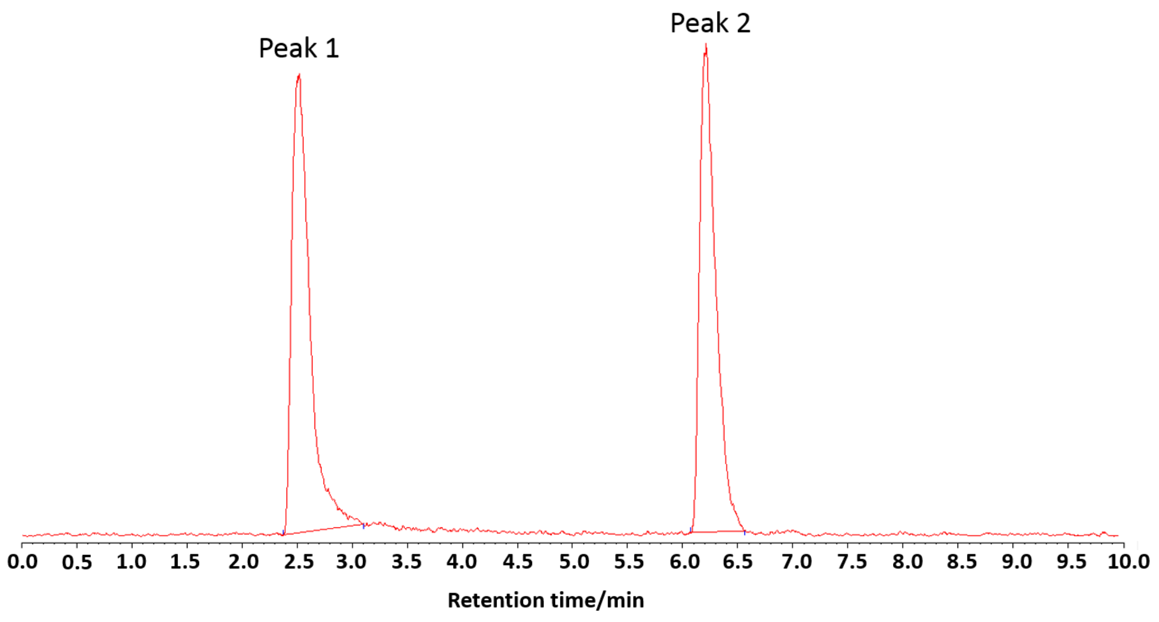

Figure S9. radio-HPLC chromatogram of the purified product eluted from a HLB cartridge and formulated in 50% EtOH/ascorbic acid solution. Peak 1: Rt = 2.58 min 45% ($^{18}F^-$). Peak 2: Rt = 6.24 min 55% ([$Ga^{18}F^{19}F_2(BnMe_2tacn)$]).

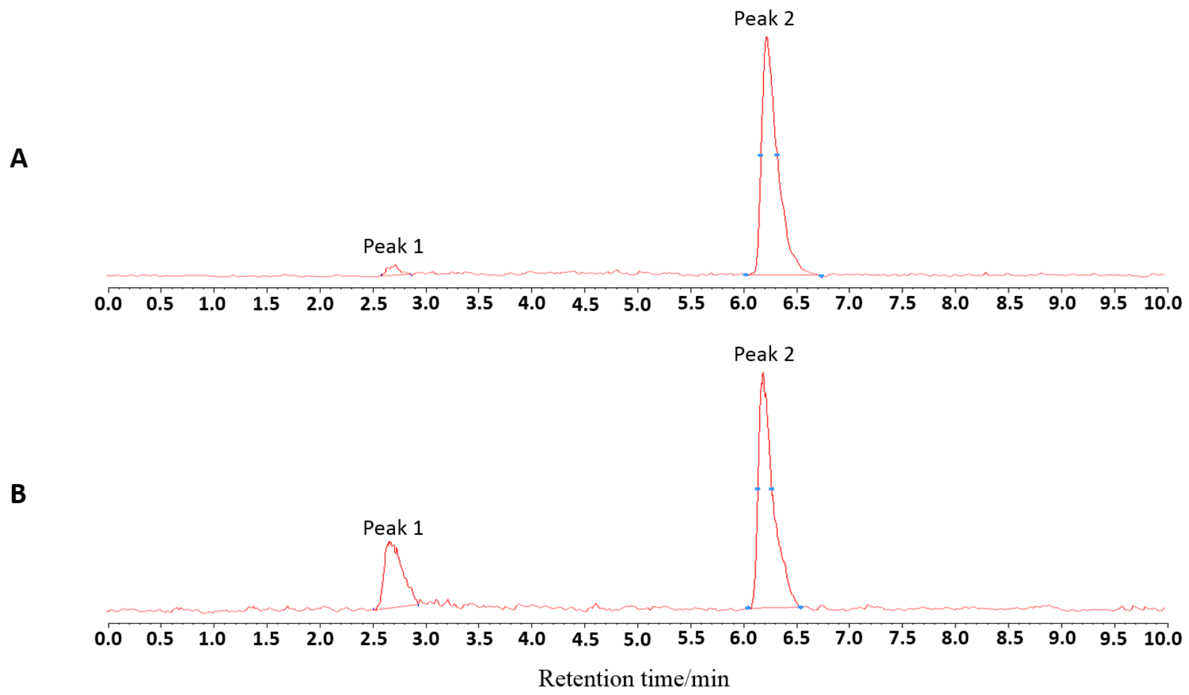

Figure S10. % RCP trend over time for the radio-product from [GaF_3($BnMe_2$-tacn)] (0.1 mg, 268 nmol) and [^{18}F]F^- in 75%/25% MeCN/H_2O at 80 °C for 10 mins., purified through a HLB cartridge and formulated in 10% EtOH/PBS pH 7.4. **A**: radio-HPLC chromatogram at t = 0; Peak 1: Rt = 2.72 min 3% ($^{18}F^-$). Peak 2: Rt = 6.21 min 97% ([$Ga^{18}F^{19}F_2$($BnMe_2$tacn)]). **B**: radio-HPLC chromatogram at t = 120; Peak 1: Rt = 2.70 min 26% ($^{18}F^-$). Peak 2: Rt = 6.21 min 74% ([$Ga^{18}F^{19}F_2$($BnMe_2$-tacn)]).

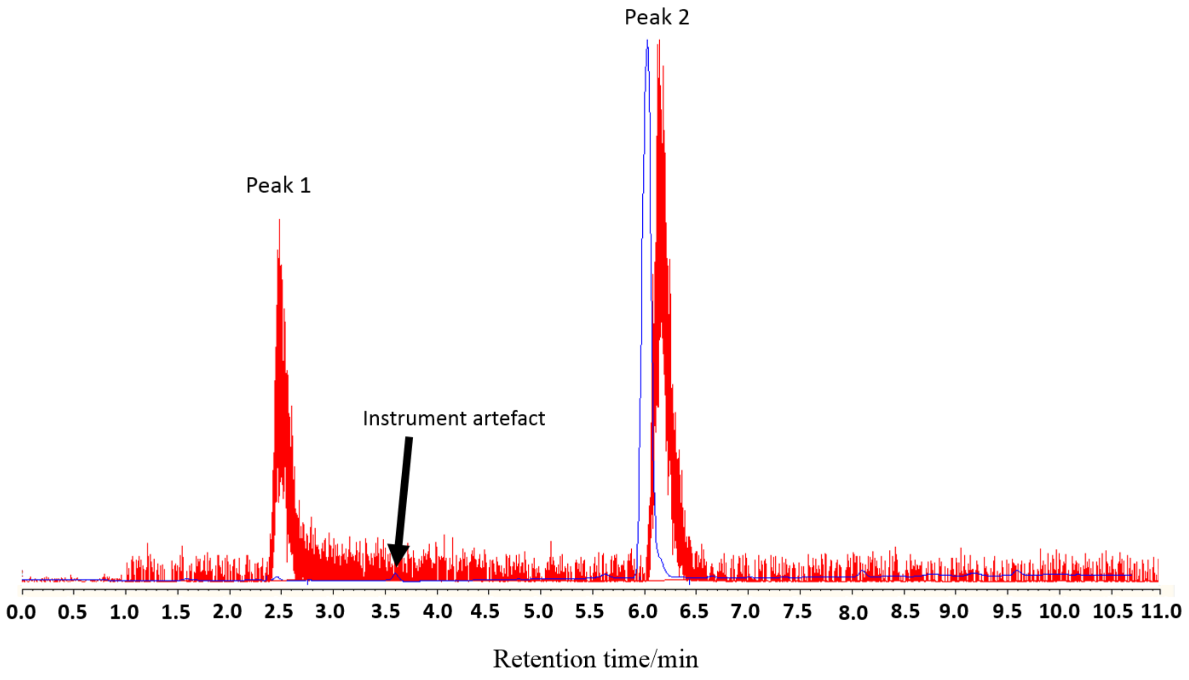

Figure S11. Radio-HPLC chromatogram (red) and the corresponding UV-trace (blue) of the crude product from radiofluorination of [GaF_3($BnMe_2$-tacn)] (1 mg, 2.68 μmol) in 75%/25% MeCN/H_2O at 80 °C for 10 mins. Peak 1: Rt = 2.51 min 33% ($^{18}F^-$). Peak 2: Rt = 6.14 min 67% ([$Ga^{18}F^{19}F_2$($BnMe_2$tacn)]). Peak 2 matches the UV-vis peak of [GaF_3($BnMe_2$-tacn)].

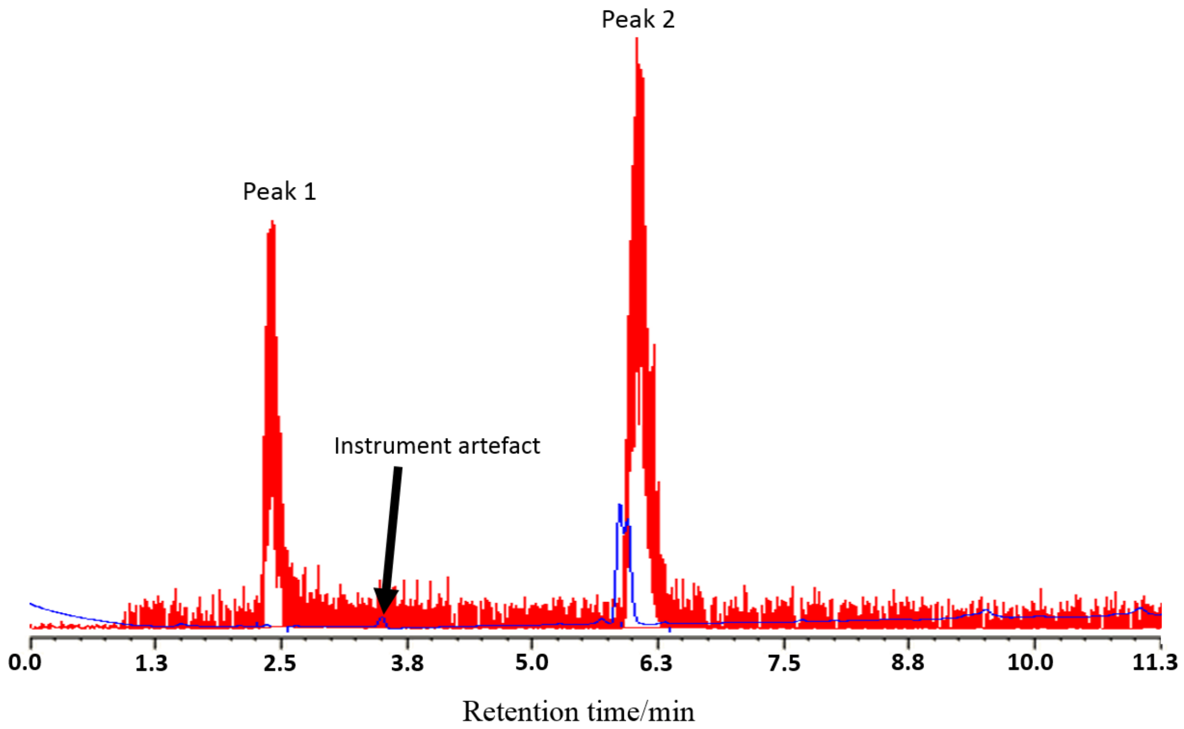

Figure S12. Radio-HPLC chromatogram (red) and the corresponding UV-trace (blue) of the crude product from radiofluorination of [GaF_3($BnMe_2$-tacn)] (0.1 mg, 268 nmol) in 75%/25% $MeCN/H_2O$ at 80 °C for 10 mins. Peak 1: Rt = 2.52 min 32 % ($^{18}F^-$). Peak 2: Rt = 6.13 min 68 % ([$Ga^{18}F^{19}F_2$($BnMe_2$-tacn)]). Peak 2 matches the UV-vis peak of [GaF_3($BnMe_2$-tacn)].

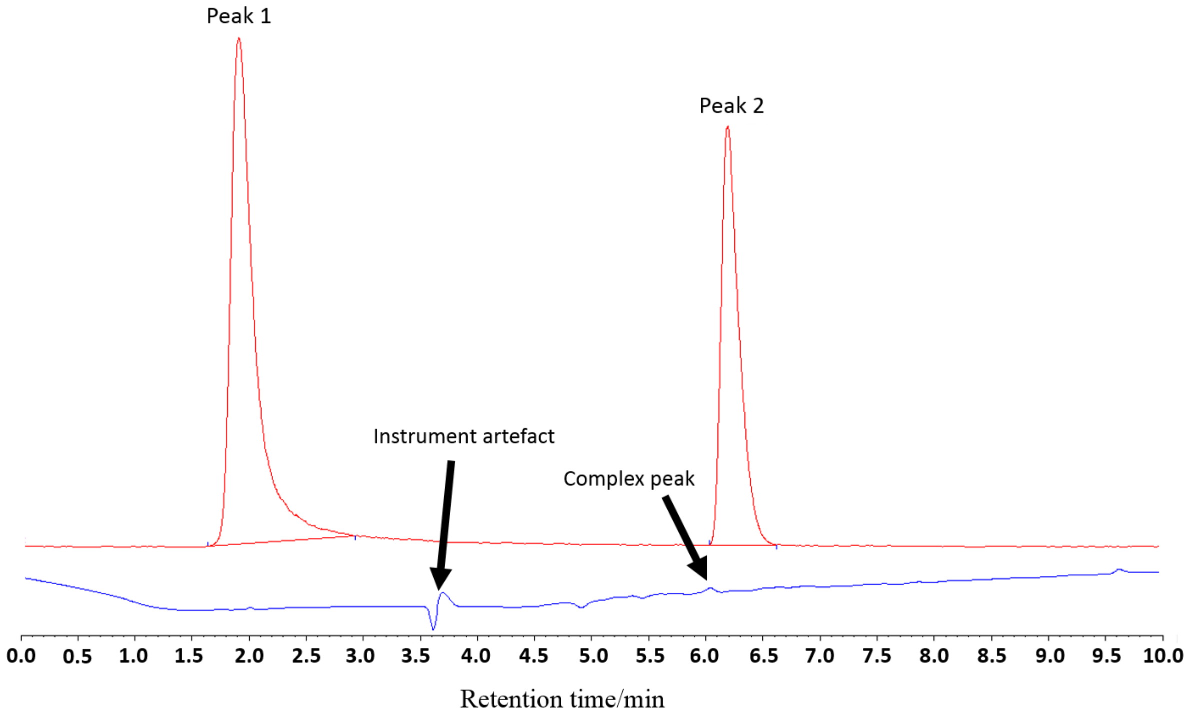

Figure S13. Radio-HPLC chromatogram (red) and the corresponding UV-trace (blue) of the crude product from radiofluorination of [GaF_3($BnMe_2$-tacn)] (0.01 mg, 27 nmol) in 75%/25% $MeCN/H_2O$ at 80 °C for 10 mins. Peak 1: Rt = 2.11 min 62% ($^{18}F^-$). Peak 2: Rt = 6.19 min 38% ([$Ga^{18}F^{19}F_2$($BnMe_2$tacn)]).

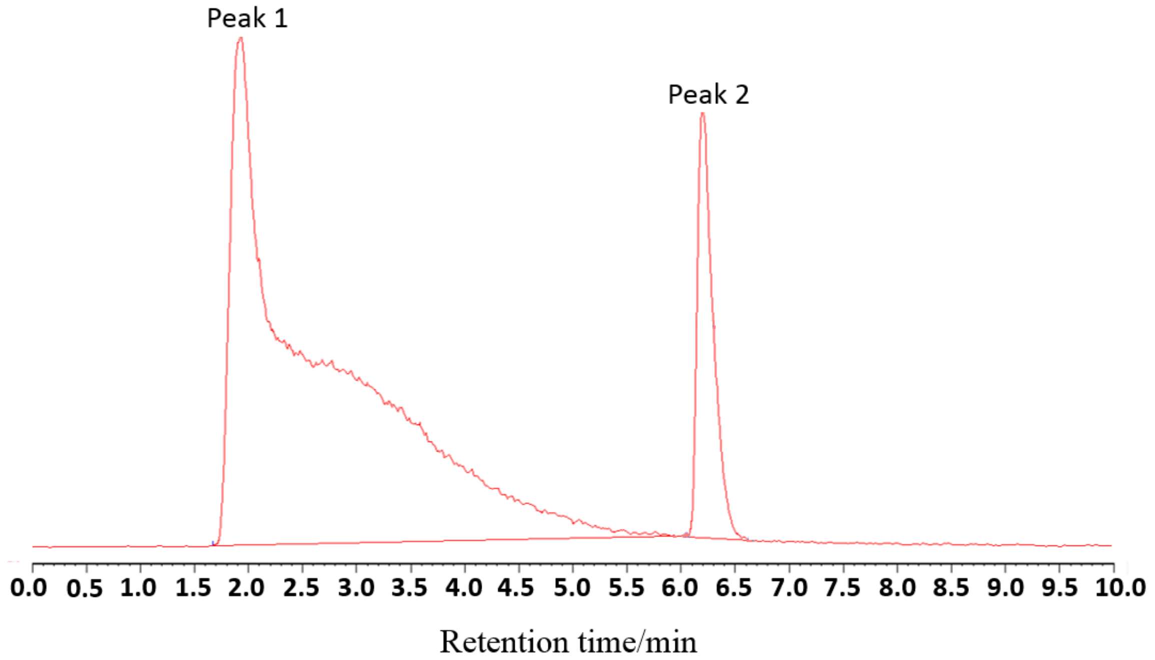

Figure S14. Radio-HPLC chromatogram of the crude product from radiofluorination of [GaF_3($BnMe_2$-tacn)] (0.1 mg, 268 nmol) in 75%/25% DMSO/H_2O at 80 °C for 10 mins. Peak 1: Rt = 2.57 min 86% ($^{18}F^{-}$). Peak 2: Rt = 6.16 min 14% ([$Ga^{18}F^{19}F_2$($BnMe_2$tacn)]).
